# Notes and Comments

**Published:** 1893-09

**Authors:** 


### Notes and Comments.[1]

FURTHER USE OF NITRATE OF SILVER.—The following recent communication from Dr. Stockwell is self-explanatory, and we hope others will experiment and report along this line of investigation.

---

[1] The assistant editor solicits contributions for this department,—new methods, new remedies and formulas, or any short practical note which may prove of value to the practitioner or student. Address 1506 Arch Street, Philadelphia.

TO THE ASSISTANT EDITOR:

"Apropos of your item on nitrate of silver in May issue of the INTERNATIONAL DENTAL JOURNAL, allow me to say that I am experimenting, thus far successfully, with its use in the treatment of putrescent pulp-canals. We have a class of molars where, for various reasons, it is nearly or quite impossible to reach the apex of one or more canals with the desired thoroughness and certainty. In short, all we can do is to do "the best we can." These are the cases that suggested to my mind the nitrate of silver treatment.

"With the rubber dam in place, I introduce into the chamber and carry as far as possible into the canals, a strong solution—fifty per cent. and upward—of nitrate of silver, afterwards sealing in the chamber a pledget of cotton saturated with the same, and let it remain for a day or two. Then fill as successfully as possible with such material as circumstances indicate. The object, of course, is to thoroughly sterilize the desired territory, and I have a theory that the action of the drug upon the dentine and the contents of the canals and tubuli is such that we have permanent antiseptic condition.

"Will others kindly experiment and report?

"C. T. STOCKWELL.

'SPRINGFIELD, MASS."

